# An Integrated Blueprint for Digital Mental Health Services Amidst COVID-19

**DOI:** 10.2196/21718

**Published:** 2020-07-22

**Authors:** Luke Balcombe, Diego De Leo

**Affiliations:** 1 Australian Institute for Suicide Research and Prevention Griffith University Brisbane Australia

**Keywords:** digital mental health, mental well-being online assessments, machine learning, automation, COVID-19, well-being services

## Abstract

In-person traditional approaches to mental health care services are facing difficulties amidst the coronavirus disease (COVID-19) crisis. The recent implementation of social distancing has redirected attention to nontraditional mental health care delivery to overcome hindrances to essential services. Telehealth has been established for several decades but has only been able to play a small role in health service delivery. Mobile and teledigital health solutions for mental health are well poised to respond to the upsurge in COVID-19 cases. Screening and tracking with real-time automation and machine learning are useful for both assisting psychological first-aid resources and targeting interventions. However, rigorous evaluation of these new opportunities is needed in terms of quality of interventions, effectiveness, and confidentiality. Service delivery could be broadened to include trained, unlicensed professionals, who may help health care services in delivering evidence-based strategies. Digital mental health services emerged during the pandemic as complementary ways of assisting community members with stress and transitioning to new ways of living and working. As part of a hybrid model of care, technologies (mobile and online platforms) require consolidated and consistent guidelines as well as consensus, expert, and position statements on the screening and tracking (with real-time automation and machine learning) of mental health in general populations as well as considerations and initiatives for underserved and vulnerable subpopulations.

## Introduction

It has been proposed that using digital health technology can strengthen our health systems [1]. Coupled with high-quality mental health research, digital innovations can aim at resolving “the potential crisis in the provision of health services to helping preserve and reconstruct a post-pandemic society” [2]. Digital mental health services delivered online and through smartphone technologies can be useful for targeted psychological interventions in communities affected by coronavirus disease (COVID-19) [3]. These provisions may fill the critical gap that practitioners are facing because of higher demand for mental health care services and the limitations of face-to-face consultations [4,5]. In this regard, the COVID-19 crisis could accelerate the use of mobile and teledigital health or at least challenge the usual standard of care [6].

## Mental Well-Being Online Assessments and Automated Analysis Via Machine Learning

Data collection and analysis must be large-scale and of high quality to address mental health needs under the current pandemic. An example of an innovative, tailored, and practical technology applied in research methods is the machine learning induction of models. This technology was applied in a study by Wshah et al [7] to predict the probability of posttraumatic stress disorder (PTSD) symptoms in patients 1 month after trauma using self-reported symptoms from data collected via smartphones. The results suggest that simple smartphone-based patient surveys, coupled with automated analysis using machine learning–trained models, can identify those at risk for developing PTSD symptoms, and thus target them for early intervention.

It has long been acknowledged by mental health practitioners that there is a need to activate all possible opportunities to offer help, including through teleassistance, to patients [8]. At-home, mental health treatments are mostly limited to telehealth, where providers remotely communicate with patients over the phone or using video [9]. Telemental health services are perfectly suited to pandemic situations, with people being given access to mental health assistance without increasing the risk of contracting infections [10]. In times of crisis, the mental health of people needs to be supported in any way possible [11]. The call for innovative and expansive solutions and broad-scale collaboration in mental health prevention and intervention delivery includes support for technology [12]. Screening for and tracking of stress and adjustment issues in the general population via simple online/smartphone-compatible surveys, coupled with automated real-time analysis using machine learning–trained models, can deliver faster and better mental health care.

## COVID-19 Mental Health Crisis: A Booster for Digital Interventions

Reliable, valid, and replicable mental health screening and tracking tools via machine learning have the potential to provide an integrated blueprint for the COVID-19 mental health response. The possibility of an integrated offering requires a strategy that brings together different areas of expertise (ie, psychology, psychiatry, emergency and general health care, human welfare, and digital innovation). There is a range of ground-breaking developments happening, with automation taking hold rapidly and consensus to broaden horizons beyond telehealth to deliver digital assets [5,13,14]. A willingness to learn, the capacity to adapt to changes, collaboration, and well-connected management can help to resolve the organizational problems that continue to hamper the large-scale development and implementation of new technologies. The tragic impact of the COVID-19 pandemic may act as a booster for a rapid growth in activities related to digital interventions.

However, the adoption of digital interventions was recommended well before the onset of the pandemic and suggested as a complementary service that functions alongside traditional treatments, rather than be their replacement [15]. An important caveat is the possible lack of access for vulnerable people needing health care [1]. The consequences of COVID-19 have emphasized the urgent need to counteract the psychological impact of the pandemic by facilitating access to psychiatric diagnosis and treatment [16] while maintaining social distancing.

Mental health services could be broadened by training unlicensed professionals such as those who work in academia as researchers or in other areas of psychology, psychiatry, or mental health that don’t require direct clinical contact with patients. Self-help interventions can be delivered through a variety of media; these have proven effective for a range of mental health problems [17] and could be further explored for whether adjustment to a remote care set-up is linked to a (more) fertile mindset to solve issues.

## Evaluation of Digital Interventions Required for Quality Assurance

Quality assurance remains problematic for online psychological services in low- and middle-income countries [18] and vulnerable populations [14]. There are no current systems known to be in place to evaluate digital mental health innovations with respect to underserved populations. International standards on service quality are essential, as are accessibility, sustainability, equity, and ethics [4,19]. Rigorous evaluations are needed to answer questions related to utility, effectiveness, confidentiality, and quality of interventions [20,21].

## Conclusion

The global potential of digital mental health in improving the accessibility and quality of mental health service provision was boosted during the COVID-19 pandemic. A recent paper by Torous et al [14] focused on the requirements for increased efforts around safety, evidence, engagement, outcomes, and implementation to increase the scalability of and access to quality digital mental health care and proposed funding, research, policy changes, training, and equity as investments that will yield ongoing returns. In response, Whelan et al [4] emphasized the need for ongoing evaluation to ensure national evidence standards, digital training for mental health professionals, and assessment of how digital health innovations can be safely and sustainably embedded in care pathways with reference to the nonadoption, abandonment, scale-up, spread, and sustainability (NASSS) framework [22].

In adopting, scaling up, spreading, and sustaining digital mental health innovations in the midst of the COVID-19 pandemic and on a long-term basis, we recommend coordinating organizational and system framework assessments with evidence-based, online/smartphone-compatible screening and tracking tools deployed with real-time automation by machine learning–trained models. The results should be presented on sustainable, connected, and geocoded digital platforms, which may be supported by unlicensed professionals in order to expand and maintain patient engagement and increase options for prevention and intervention. A patient is thereby presented with recommendations for mental health care services including options for self-care and practitioner-led care with focus on value and capabilities. We also recommend suitably qualified content on mental health to be disseminated and accessed via digital workspaces with dashboards to build efficiency, consistency, and transparency from a single, global location.

Scalable screening and tracking tools should be implemented in a hybrid model of care combining face-to-face, telehealth, and digital-health approaches. Machine-readable paper copies or non–smartphone-based messaging could be adapted as solutions where there is a lack of access to the technological resources required for engagement in digital mental health services. An added value of digital mental health is that it may be designed for automated thematic and metadata review, traceability for quality assurance, and assignment of responsibility for identified cases of mental ill health. Existing telepsychiatry guidelines (including other relevant digital technologies) should be considered [23] for a consistent and consolidated knowledge management strategy for integrated services. Consensus, expert, and position statements are required from psychiatrists, psychologists, and academic researchers on the individual, cultural, and environmental factors that affect the well-being of the patient with suggestions for brief, valid and reliable screening and tracking surveys for the prevention and treatment of mental health symptoms and disorders. A general population version is needed with specifications for vulnerable subpopulations such as children, college students, domestic violence victims, frontline heath care workers, low socioeconomic groups, athletes, people with mental health disorders, and the elderly.

## Abbreviations

COVID-19: coronavirus disease
NASSS: nonadoption, abandonment, scale-up, spread, and sustainability
PTSD: posttraumatic stress disorder
